# Application of Machine Learning to Auto-Code Injury Data in the e-CHIRPP System: Development and Evaluation Study

**DOI:** 10.2196/69143

**Published:** 2025-12-17

**Authors:** Shamir N Mukhi, Steven R McFaull, Wendy Thompson, Tim Beattie

**Affiliations:** 1 Canadian Network for Public Health Intelligence, Public Health Agency of Canada, 9700 Jasper Ave, Edmonton, AB, T5J 4C3, Canada, 1-204-771-4698; 2 Canadian Hospitals Injury Reporting and Prevention Program, Centre for Surveillance and Applied Research, Public Health Agency of Canada, Ottawa, ON, Canada

**Keywords:** injury, poisoning, surveillance, machine learning, auto-coding, public health, informatics

## Abstract

**Background:**

The Canadian Hospitals Injury Reporting and Prevention Program (CHIRPP), a Public Health Agency of Canada program established in 1990, is an injury and poisoning sentinel surveillance system that collects and analyzes data on injuries to individuals who are seen at the emergency departments of numerous pediatric and general hospitals in Canada. Since its inception, the program has collected over 4 million records. The program’s surveillance activities have contributed substantially to evidence-based decision-making to reduce injuries, support research, and establish preventive safeguards to protect the health and safety of Canadians. Patients presenting at participating hospitals are asked to complete a data collection form capturing the causes and circumstances contributing to the injury or poisoning event. Using this text, hospital and program staff have traditionally coded numerous surveillance variable codes manually for subsequent analysis within e-CHIRPP, the program’s purpose-built analytical application on the Canadian Network for Public Health Intelligence public health informatics platform. Manual coding of this complex data is administratively burdensome and results in a significant time lag in the availability of important surveillance findings.

**Objective:**

With the initial goal of achieving a preliminary stage of implementation, the objective was to establish the capability to achieve enhanced timeliness of surveillance findings within a process of adaptability and continuous improvement by applying machine learning to auto-code injury data based on patient narratives.

**Methods:**

The research, development, and implementation of machine learning and auto-coding within the e-CHIRPP system were led by the Canadian Network for Public Health Intelligence team in collaboration with the CHIRPP program team. Data were extracted from e-CHIRPP and prepared for training, and candidate algorithms well suited for classification and supervised learning were initially assessed. Subsequently, 1 algorithm was chosen for further assessment based on initial accuracy, prediction confidence, and training time. The chosen algorithm was then further assessed in 2 stages, again using e-CHIRPP extracts: first, for a 2-year data set and then again for a 7-year data set. The sources of inaccuracies were investigated with a view to informing the refinement of the overall process and establishing ongoing adaptability and continuous improvement.

**Results:**

Auto-coding of injury variables showed a high level of accuracy in most cases when compared to variables previously coded manually. Importantly, insights were also gained into the sources of observed inaccuracies and measures to foster ongoing refinement of the process.

**Conclusions:**

The application of machine learning and auto-coding shows strong potential to benefit surveillance activities across various public health disciplines, yielding near real-time availability of intelligence, reduced administrative workload, continuous improvement, and adaptability to database growth and change.

## Introduction

### Background

Injuries have a significant impact on people, institutions, and the economy in Canada. In 2021, a total of 24,034 Canadians died due to intentional and unintentional injuries, including poisonings. Unintentional injuries were the third leading cause of death in 2021, accounting for 6.2% of all deaths [1]. In addition to the lives lost, injuries have a negative impact on the economy due to lost productivity as well as increased health care costs. It has been estimated that injuries cost approximately CAD $29.4 billion for the Canadian economy in 2018, including CAD $20.4 billion in health care costs and approximately CAD $9 billion in indirect costs [2]. To address the incidence of injuries and develop effective prevention strategies, timely and accurate information is needed to foster a clear understanding of their causes, the circumstances in which they occur, and the populations impacted, all of which can be captured through systematic collection of injury data [3].

### The Canadian Hospitals Injury Reporting and Prevention Program

The Canadian Hospitals Injury Reporting and Prevention Program (CHIRPP), a Public Health Agency of Canada program established in 1990, is an injury and poisoning sentinel surveillance system that collects and analyzes data on injuries to individuals who are seen at the emergency departments (EDs) of numerous pediatric and general hospitals in Canada [4]. Since its inception, CHIRPP has collected over 4 million records, each capturing important details, such as how the injury happened, what the injured person was doing, circumstances and factors contributing to the incident, when and where it happened, as well as the age and sex of the injured person.

These surveillance activities have allowed CHIRPP to contribute substantially to evidence-based decision-making to reduce injuries, support research, and establish preventive safeguards. CHIRPP information has supported regulations on cribs, cradles, and bassinets [5]; magnetic toys [6]; vaping product labeling and packaging [7]; corded window coverings [8]; and safe storage of detergent packets [9]. The data have informed jurisdictional changes to all-terrain vehicle driving age [10] and changes to building codes for window heights in multistory complexes. In addition, the data have revealed injuries related to bath seats and informed safety measures related to children’s play spaces and equipment, and sporting rules. Reports and publications have been produced on topics such as opioid-related ED visits before and during COVID-19 [11], self-harm during COVID-19 [12], e-cigarettes and vaping substances [13], as well as sports-related injuries and concussions [14].

Data collection is made possible through this longstanding collaborative partnership between participating hospitals and the CHIRPP program. In 2011, CHIRPP realized significant enhancements to the timeliness, flexibility, and adaptability of their surveillance tools through the implementation of the custom-developed e-CHIRPP system, a secure, web-based surveillance and data management technology on the Canadian Network for Public Health Intelligence (CNPHI) scientific public health informatics platform [15]. CNPHI and CHIRPP worked in collaboration to advance an innovative, dynamic code management system, which was developed and integrated within the initial release of e-CHIRPP to streamline how information was collected on the forms and aid in the adaptability of coding. This enabled the CHIRPP program team to maintain codes according to changing needs while aligning with associated coding rules and versioning, supporting improved coding and data integrity. While auto-coding of CHIRPP data was always a part of the proposed vision for CNPHI, e-CHIRPP established an important foundation for its future implementation.

Since then, there have been over a million injury cases captured in e-CHIRPP, yet despite the efficiencies gained, there are still manual tasks required of hospital staff and the CHIRPP program team. Currently, when a patient presents at a participating ED, the patient or caregiver is requested to complete the first part of a data collection form capturing the circumstances of the injury and how it occurred. Afterward, the attending physician or other hospital staff complete the second part of the data collection form, describing the nature of the injury, the treatment received, and any information on substance use, if applicable. Hospital staff code approximately half of the CHIRPP variables, creating draft cases that require further review by CHIRPP. After the draft cases are reviewed, coding is finalized by CHIRPP personnel. In general, coding that requires interpretation is completed by program staff, with the goal of describing each case as fully and consistently as possible, before being finalized.

### Rationale

To accurately capture the wide array of causes and circumstances surrounding injury and poisoning events, CHIRPP uses a large number of codes that enable detailed analysis and intelligence generation via e-CHIRPP. For example, the variable *Intent* (capturing whether the injury was intentional or unintentional) has 8 codes to classify the information related to intent that corresponds to the *International Classification of Diseases, Tenth Revision*, coding, established by the World Health Organization. Additional examples include the variable *Location,* which uses 57 codes to accurately describe where the injury happened, and the variables *Direct cause* and *Factors 1‐5*, each using the same 861 codes, thereby offering many layers to describe factors contributing to the injury. These and other variables are currently coded manually based on the patient narrative. Due to the complexity of CHIRPP data, the large number of codes, the large number of patients, and the ongoing possibility of adding new partner hospitals, there is an administrative burden resulting in a significant time lag between a patient’s presentation at a hospital and the coding of the form used to complete a record on e-CHIRPP. Multiple studies in the past have also reported that manual coding of injury-related information and factors based on a free-text narrative is a time-consuming process [16]. The initial auto-coding work undertaken by CNPHI and CHIRPP commenced in 2019. At that time, the most recently available coded data were from 2017, a lag of 2 years. Table 1 presents the 18 CHIRPP variables and the number of codes used for each.

**Table 1.** Overview of CHIRPP^a^ variables and related codes.

CHIRPP variable	Description	Number of codes
Indoor/outdoor^b^	Indicates whether the injury occurred indoors or outdoors.	3
Location	Describes where the injury generally occurred. It is used in conjunction with the area to further specify the room or named place where the injury/poisoning event occurred.	57
Area	Used to further specify within a general location where the injury/poisoning event occurred.	51
Context	Describes what the patient was doing or what activity they were engaged in when the injury/poisoning event occurred.	48
Vehicle type	The type of boat or motorized transport vehicle occupied by the patient in the injury/poisoning event.	13
Vehicle seating position	The vehicle seating position of the patient in the boat or motorized transport vehicle captured in vehicle type.	21
Direct cause^c^	The factor for which the most severe injury is attributed.	861
External cause	Describes both the intent and primary diagnosis (mechanism) of injury using the *International Statistical Classification of Diseases and Related Health Problems, 10th Revision*.	28
Sports/recreation	Any activity that requires physical exertion beyond baseline levels or an activity that is physical and uses a specific piece of equipment or is a physical game with a specific purpose.	120
Organized sport or activity	Indicates if the sport or physical activity that the patient was participating in at the time of the injury included coaches, instructors, or officials.	4
Safety equipment used	Identifies if safety equipment was used by the patient during the injury event.	3
Substance use	Indicates if illicit drugs, medications, alcohol, or other substances, either by the patient or another person, directly or indirectly caused the injury.	4
Intent	Describes the deliberate nature of the reported injury or poisoning by a human person. Injury or poisoning by the patient or another person is intentional when the injury or poisoning was the expected outcome.	8
Factors 1 to 5^d^	Inanimate objects, substances, and living things that directly or indirectly caused (contributed to) the injury/poisoning event.	861 each

a CHIRPP: Canadian Hospitals Injury Reporting and Prevention Program.

b Indoor/outdoor is binary; however, a third code captures instances where this is *unknown*.

c The direct cause code is chosen from the selection of factor codes. Coders first enter a factor code for the direct cause of the injury/poisoning corresponding to the most severe injury or poisoning, if known, and then may enter up to 5 additional factor codes, which also contributed to the injury/poisoning.

d Factors 1 to 5 provide additional layers to describe factors contributing to the injury.

Accordingly, there is a strong rationale to explore the applicability of machine learning and auto-coding of injury data with a view to greatly increasing the timeliness of intelligence arising from CHIRPP’s surveillance. The approach can potentially assist in coding variables automatically, nearly in real time, as an innovative alternative to manual coding. In addition, the approach minimizes the need for human intervention, compared to manual coding, as the algorithm would be trained using predefined training data [17].

### Objective

The objective of this initiative was to apply and assess the capability of machine learning to complete timely and accurate auto-coding of defined variable codes for injury and poisoning events in e-CHIRPP based on information provided by patients or caregivers and to identify steps leading to preliminary implementation and ongoing refinement of the approach.

## Methods

### Overview

When a patient presents at a participating CHIRPP ED with an intentional or unintentional injury or poisoning, information is collected with the goal of fully describing the injury event and its context and location. For the purpose of this initiative, we used a machine learning algorithm to explore the feasibility of auto-coding 18 CHIRPP variables based on case narratives.

### Ethical Considerations

Each hospital in the CHIRPP program has undertaken its own ethics review; no additional ethics review was required for this study. The data were drawn from an existing deidentified surveillance database and, therefore, anonymized. All methods were performed in accordance with the relevant guidelines and regulations.

### Data Processing

Historical data from e-CHIRPP were extracted from the system. Two steps were performed, as described below.

Text tokenization was performed for the input variables. This is a process of dividing texts into words or smaller subtexts, determining the *vocabulary* of the data. This is like identifying various keywords of relevance that can be recognized by the algorithm and labeling the keywords with the appropriate codes.

This was followed by a feature selection process for the input variables. This is a process of removing irrelevant information that does not contribute to coding and prepares the dataset to be used for algorithm training.

### Algorithm Selection

#### Overview

Numerous machine learning algorithms are available for an array of applications. The selection is not a question of which algorithm is best; it is a question of which is best suited for a particular type of application based on the dataset and the task at hand.

For the purpose of assessing algorithms to be used with CHIRPP data, numerous candidate algorithms were initially selected based on their suitability.

#### Classification

For application with CHIRPP data, machine learning is applied to predict which category the input data belongs to, whether the variables are binary (a or b), or multiclass (numerous).

#### Supervised Learning

This is a process of *teaching* an algorithm with labeled data (tokenized text) to prepare it for making predictions with real unforeseen data.

While a machine learning algorithm can be thought of as a computer-coded procedure for recognizing specific attributes or patterns within a dataset, a machine learning model comprises the output of an algorithm trained with labeled data and prepared for making predictions on unforeseen data [18].

The k-fold cross-validation method was used to assess the algorithms. This procedure involved randomizing the labeled data and then dividing it into *k* groups (folds) of approximately equal size. In each of a repeated series of trials, all but one of the folds are used as training data while one fold is used as a test data sample on which the algorithm makes predictions. Each fold is used once as the test data sample until the process is completed [19].

Evaluation of the algorithms was done using the following criteria: (1) accuracy (percentage of records that were classified correctly), (2) average confidence of all samples coded correctly (prediction confidence denoted by the model), (3) average confidence of all samples coded incorrectly (prediction confidence denoted by the model), and (4) training time (amount of time taken to train the model).

The purpose of this evaluation was to compare different machine learning algorithms and to select a single algorithm best suited for subsequent analysis. Multilayer perceptron (MLP) was selected as the preferred algorithm.

### Evaluation of the MLP Algorithm

As a first step, data from e-CHIRPP for 2016 to 2017 were extracted from the system (316,803 cases). After removal of any draft cases, 80% of the remaining data were used as a training set to train the selected algorithm, and the remaining 20% of the data were used to test the algorithm. Using the injury event description or narrative, the auto-coding accuracy for each of the 18 CHIRPP injury variables was assessed.

To identify potential reasons for errors and misclassifications, a subset of samples that were misclassified was randomly selected and assessed manually.

The e-CHIRPP data contain codes that are used to indicate variables that are either unknown, irrelevant, or unable to be determined by the coder. Examples of these codes are *0IO: Missing* for the variable *indoor/outdoor* and *99A: unknown area* for the variable *area.* We refer to these codes as *null codes*. Simulations were repeated with null codes removed to assess the impact of potential *noise* removal and assess if the accuracy would improve.

This was followed by the extraction of 7 years of previously coded injury data from e-CHIRPP for 2011 to 2017 (976,249 cases). Similarly, after removal of any draft cases, 80% of the data were used as a training set to train the algorithm, and the remaining 20% were used to test the algorithm. The same analysis done on the initial 2 years of data was repeated on the 7-year dataset to determine whether an increase in the number of records had any impact on the accuracy of the auto-coding of the variables.

Given that the work of implementing machine learning and auto-coding was underway in 2019, the most recently coded data available at that time were from 2017 and earlier. It should be noted, however, that the arrival of the COVID-19 pandemic in 2020 impacted the pace of this initiative.

## Results

### Outcome Measures

The accuracy of auto-coding achieved by the algorithm was assessed by comparing the auto-coding results to the manually coded data, with % accuracy serving as an outcome measure based on the level of agreement between the two.

### Primary Outcomes

Overall, we found that machine learning and auto-coding of injury variables showed a high level of accuracy in most cases when compared to variables previously coded manually. Importantly, we also gained insight into the potential sources of inaccuracies, as discussed in the secondary outcomes. Table 2 summarizes the primary outcomes achieved in all trials.

The % accuracy for auto-coding was highest for the variables *indoor/outdoor, substance use,* and *intent,* in general. This finding was consistent for both the 2- and 7-year datasets.

The accuracy was lowest for the variables *vehicle seating position* and *factors 1 to 5* for both datasets. We also found that the accuracy increased for all variables when null codes or missing values were removed, except for the variables *vehicle type* and *factors 4 and 5.*

In addition, when more data were used (7 years), the change in auto-coding performance was insignificant for most of the variables.

We also observed that the auto-coding accuracy progressively diminished in the results for *factor 1* through *factor 5.*

**Table 2.** Canadian Hospitals Injury Reporting and Prevention Program variables tested, number of codes for each variable, and % accuracy in auto-coding for the 2- and 7-y datasets.

Variables	Number of codes	2-y dataset including missing and null values (%)	2-y dataset missing/null values removed (%)	7-y dataset missing/null values removed (%)
Indoor/outdoor	3	89.96	95.32	95.36
Location	57	82.23	88.85	87.42
Area	51	87.19	93.33	91.25
Context	48	77.33	79.28	79.96
Vehicle type	13	98.84	84.91	85.18
Vehicle seating position	21	65.02	69.60	73.14
Direct cause	861	71.28	77.91	79.09
External cause	28	87.60	90.74	90.63
Sports/recreation	120	93.36	93.45	94.12
Organized sport or activity	4	85.30	94.60	92.54
Safety equipment used	3	79.84	92.40	92.40
Substance use	4	98.67	98.77	98.89
Intent	8	98.36	98.90	99.20
Factor 1	861	70.83	72.71	74.13
Factor 2	861	47.90	51.15	53.04
Factor 3	861	38.01	41.70	41.27
Factor 4	861	40.74	25.93	28.74
Factor 5	861	50.00	33.33	33.33

### Secondary Outcomes

We manually assessed the cases that were incorrectly coded by the model to identify potential reasons for the misclassifications.

Some keywords extracted from the narratives did not have sufficient information at the time of coding. The narratives for some records were rich and consisted of many words or a few sentences, whereas some records had very limited narratives. This exercise helped to emphasize the importance of capturing a sufficient narrative from the patient or caregiver and hospital staff.

Some records had confusing narratives. For instance, for the variable *“indoor/outdoor*,” there were some words in the narrative indicating that the injury occurred outdoors and some words indicating that the injury occurred indoors.

There were also inconsistencies in the historical, manually coded data used for training the algorithm. For instance, some coders coded injuries that occurred at the entrance of a building as indoors, whereas others coded them as occurring outdoors. Given the numerous codes related to some variables, this suggests that to some extent, the machine learning model revealed possible inconsistencies among human coders.

On investigating the observation that the auto-coding accuracy results progressively diminished from *factor 1* through *factor 5,* we observed an increasing scarcity in the use of these codes as well as an increasing proportion of unpopulated data from *factor 1* through *factor 5* in the previously coded data.

## Discussion

### Strengths

Machine learning showed a promising capability for auto-coding complex injury and poisoning data derived from the free text narratives provided by patients or caregivers and hospital staff, with a high level of accuracy. The findings from this experience can serve to inform the successful implementation and ongoing refinement of the approach in this and other areas of public health surveillance and response.

The application of machine learning lends itself well to continuous improvement. With insights gained from the preliminary implementation of auto-coding and with supporting enhancements to the program’s custom-built surveillance and analytical application on CNPHI, e-CHIRPP, the following features have been enabled: (1) randomized case selection for manual quality assurance verification of coded cases, with the rate of case review to be determined by the CHIRPP program; (2) flagging of cases coded with high confidence to recover examples of strong narratives to share with participating CHIRPP hospital staff for training and awareness purposes; and (3) flagging of cases of low confidence for manual review and to inform a cycle of algorithm retraining.

### Limitations

#### Challenges Posed by the Large Number of Codes

We found that overall, the % accuracy for variables with a large number of codes, such as *factors 1 to 5* and *direct cause* (each using 861 codes), was lower than that of other variables. One possible explanation for this is that rarely used codes probably did not appear frequently enough in the extracted data. As a result, the algorithm did not have sufficient opportunity to learn the rarely used coding from the records [20].

Another possible explanation for low % accuracy is that due to the large number of codes, there is a higher likelihood of coding errors or inconsistencies among human coders. This would also impact the data used for algorithm training and testing. On the contrary, all the variables with less than 10 codes had an accuracy of greater than 90% for both the 2- and 7-year datasets without null codes, which supports the above reasoning.

#### Null or Missing Codes

The % accuracy increased for most of the variables when null and missing codes were removed. From a practical standpoint, it is undesirable for the algorithm to learn from data that are labeled as *unknown*. For example, the accuracy for auto-coding the variables *vehicle type* and *factors 4 and 5* decreased after null codes were removed because, in most cases, null codes appeared in the training data for these variables. This resulted in a significant decrease in the sample size available for algorithm training. The additional *factor* variables are available to describe cases involving more complex, multifactor circumstances contributing to an injury/poisoning event. While such cases are rare, it is important that the additional *factor* variables are available when needed, even if they are often not used. An increasing occurrence of null or missing codes in *factors 3* to 5 is an expected attribute of the data. For example, the CHIRPP team looked at a sample of coded cases (n=1,583,274) and found that, taken together, *factors 3* to 5 were used in less than 1% of the records. As a result, the remedy for null or missing codes aligns with that proposed for scarcely used codes, discussed below.

#### Scarcely Used Codes and Imbalanced Algorithm Training Data

When more data were used to make predictions (7 years), the change in performance for most variables was insignificant, which was initially an unexpected result. While it has been shown that the accuracy of auto-coding increases as the size of the training dataset increases, the incremental increase in accuracy eventually plateaus after a certain point, despite further increases in the size of the dataset [21]. However, rather than reaching a plateau, we suggest our results tend to highlight the degree of scarcity in the use of some codes because the use of additional data had little impact on accuracy, particularly for the variables *direct cause* and *factors 1 to 5*, which all use the same 861 codes. While additional volumes of training data are recommended for further rounds of training and assessment, future remedies to address scarcely used codes could also include the use of artificial training data to target the most scarcely used codes not encountered in training data.

One of the biggest challenges we faced was with imbalanced datasets where some codes appeared significantly more frequently than others. Some variables (*vehicle type*, *substance use*, and *intent*) have highly skewed datasets where >90% of all cases have *dominant* codes. The drawback of this is that if a certain variable is highly skewed, then a model could simply always predict the dominant codes, and it would still score a high accuracy, while remaining unsuccessful in learning the nondominant codes. We suggest that the best way to deal with this is to rebalance the dataset by removing some of the dominant codes to achieve a dataset with 50% dominant and 50% nondominant codes for training purposes. This may have the effect of lowering the overall accuracy slightly but can significantly increase the accuracy of auto-coding nondominant codes. Various approaches to rebalancing datasets continue to evolve in the area of machine learning, which can be incorporated as a part of the continuous improvement of the overall process [22].

#### Subjectivity in Recalling or Interpreting Details

Information provided in patient narratives may be impacted by subjectivity, varying levels of detail, or differing interpretations among coders. This may partially explain the low initial results for accuracy in auto-coding the variable *context,* for example. There is a potential that inconsistencies are introduced at these stages that have a subsequent impact on algorithm training and the resulting accuracy of auto-coding.

#### Data Years Used and Pandemic-Related Impacts

Although our work commenced in 2019, using the most recently available coded data from 2017 and earlier, the project was impacted by the arrival of the COVID-19 pandemic. While the objective of reaching a preliminary stage of implementation of auto-coding injury data was achieved, further stages of refinement and validation have been informed by this initial experience.

#### A Human Resource Is Still Required

Auto-coding using machine learning is not perfect and does not fully eliminate the need for a human resource. In the future, a blended approach could involve human investigation of auto-coding results of low confidence or those flagged for attention by the model and verification of samples of auto-coded cases. Manual investigation and coding could correct such instances, supporting ongoing refinement of the process.

### Conclusions

This innovation has demonstrated the successful preliminary implementation of a solution to address the administrative burden and time lag associated with the manual coding of large quantities of complex surveillance data. Other public health disciplines could also benefit from the application of machine learning to realize near real-time availability of surveillance intelligence, fostering improved timeliness of actions and decisions to protect public health. Importantly, the process readily lends itself to ongoing adaptability and continuous improvement, offering capabilities to accommodate new or changing information fields and address sources of inaccuracy. While the objective of reaching a preliminary stage of implementation was achieved, challenges related to the collection and auto-coding of complex injury data have been characterized to inform future steps for the partners.

A strategy of ongoing verification and process review is required to ensure optimal accuracy and continuous improvement of the approach. Quality assurance and verification activities need to be customized on a program-specific basis. A human resource is still required to oversee and maintain the process. A surveillance and analytical application suitable for a program’s needs, as in the case of CHIRPP’s e-CHIRPP system on CNPHI, provides an important, complementary foundation that is instrumental in supporting continuous improvement.

Insights gained from this preliminary stage of implementation will position the partners to embark on the next steps, leading to an envisioned state where time-consuming human coding can be replaced with auto-coding supported by a rigorous strategy of quality assurance, validation, and refinement. This could include the targeted review of records below set confidence levels and randomized review of a set percentage of coded cases on an ongoing basis.

Despite some of the complexities encountered, there are many plausible benefits of auto-coding CHIRPP variables using machine learning. These benefits extend to other public health surveillance disciplines as well and may include the following:

- *Timely availability of intelligence to identify and respond to public health risks and emerging issues:* Auto-coding using machine learning could potentially provide comprehensive information in near real-time, largely eliminating lag and enabling more timely responses to protect public health.
- *Accuracy of the coding:* Algorithms can potentially improve the accuracy of the coded data by decreasing human error and eliminating inconsistencies among coders, resulting in more precise surveillance intelligence.
- *Reduction in workload for coders:* Manual coding based on a free text narrative, especially for large quantities of complex data, is very resource intensive, leading to a heavy workload for coders. Auto-coding could reduce this workload, save time, and potentially increase their productivity.
- *Database growth and addition of variables:* Manual coding becomes more challenging and complex as a system grows and new variables or codes are added. Machine learning algorithms could adapt to such growth with minimal challenges.
- *Resource allocation and program planning:* Intelligence from a completely coded database could support program planning, resource allocation, and research while informing preventive strategies to protect public health.

## Abbreviations

CHIRPP: Canadian Hospitals Injury Reporting and Prevention Program
CNPHI: Canadian Network for Public Health Intelligence
e-CHIRPP: the purpose-built analytical public health informatics application used by the CHIRPP team
ED: emergency department
